# Multiparameter behavioral profiling reveals distinct thermal response regimes in *Caenorhabditis elegans*

**DOI:** 10.1186/1741-7007-10-85

**Published:** 2012-10-31

**Authors:** Rajarshi Ghosh, Aylia Mohammadi, Leonid Kruglyak, William S Ryu

**Affiliations:** 1 Lewis-Sigler Institute for Integrative Genomics, Department of Ecology and Evolutionary Biology, Princeton University, Washington Road, Princeton, NJ 08544, USA; 2 Department of Physics, University of Toronto, St George Street, Toronto, Canada; 3 Howard Hughes Medical Institute, Princeton University, Washington Road, Princeton, NJ 08544, USA; 4 Donnelly Centre, University of Toronto, College Street, Toronto, Canada

**Keywords:** Nociception, dimensionality reduction, ethology, thermal sensation

## Abstract

**Background:**

Responding to noxious stimuli by invoking an appropriate escape response is critical for survival of an organism. The sensations of small and large changes in temperature in most organisms have been studied separately in the context of thermotaxis and nociception, respectively. Here we use the nematode *C. elegans *to address the neurogenetic basis of responses to thermal stimuli over a broad range of intensities.

**Results:**

*C. elegans *responds to aversive temperature by eliciting a stereotypical behavioral sequence. Upon sensation of the noxious stimulus, it moves backwards, turns and resumes forward movement in a new direction. In order to study the response of *C. elegans *to a broad range of noxious thermal stimuli, we developed a novel assay that allows simultaneous characterization of multiple aspects of escape behavior elicited by thermal pulses of increasing amplitudes. We exposed the laboratory strain N2, as well as 47 strains with defects in various aspects of nervous system function, to thermal pulses ranging from ΔT = 0.4°C to 9.1°C and recorded the resulting behavioral profiles.

**Conclusions:**

Through analysis of the multidimensional behavioral profiles, we found that the combinations of molecules shaping avoidance responses to a given thermal pulse are unique. At different intensities of aversive thermal stimuli, these distinct combinations of molecules converge onto qualitatively similar stereotyped behavioral sequences.

## Background

An organism's environment is characterized by temporal and spatial fluctuations of temperature. The ability of the nervous system to elicit appropriate behavioral responses to a range of thermal stimuli is critical for the animal's survival. At the molecular level, the perception of different temperature ranges, at least in mammals, is determined by the activation thresholds of relevant neurons set by distinct combinations of transient receptor potential (TRP) and potassium channels [1,2]. However, beyond these thermosensors, the behavioral, neural and molecular correlates of the transformation of temperature perception to behavioral outputs are less well understood.

The nematode *C. elegans *is an ideal model system for uncovering the molecular and cellular bases of perception of a variety of thermal stimuli [3-5]. Depending on the nature of the thermal stimulus, *C. elegans *displays distinct behaviors. During thermotaxis it can discriminate temperature differences of approximately 0.05°C [6]. At the other extreme, temperatures approximately 16°C above the baseline elicit a stereotypical avoidance behavior in this organism [7-10]. Although much is known about the neuronal and molecular bases of thermotaxis, the neurogenetic basis of the high temperature nociception is largely unknown, and only a few studies have examined intermediate thermal stimuli [3,9,11]. Most studies of noxious avoidance behavior have focused on a single aspect of the behavioral response, such as the response latency or the fraction of animals responding. However, several components of the motor output change in a coordinated manner when an animal is exposed to noxious stimuli [7]. For a comprehensive understanding of the neurogenetic basis of the behavioral response elicited by thermal pulse stimuli, it is necessary to measure multiple aspects of behavior that change as a consequence of thermal stimuli. Consideration of the multidimensional nature of a behavioral response will provide a better understanding of the role of a given molecule in shaping the behavior.

Here, we developed a high-content assay that allowed us to quantify multiple aspects of the avoidance response of animals exposed to thermal pulse stimuli of different intensities. By analyzing the responses of 47 strains defective in various aspects of nervous system function in the laboratory strain (N2) background and comparing their behavior with the N2 strain, we discovered multiple noxious temperature regimes in *C. elegans *defined by distinct combinations of molecules that converge onto a qualitatively similar stereotyped avoidance response.

## Results

### A quantitative assay to characterize responses to thermal pulses of different amplitudes

**Figure 1:**
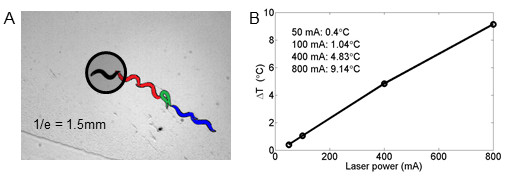
**Thermal pulse induced escape behavior**. **(A) **Thermal avoidance response. An infrared (IR) laser with a beam width larger than the worm body (1/e = 1.5 mm) heats the entire worm. The worm typically will response with a reversal (red), perform an omega turn (green), and then move forward (blue). **(B) **Temperature increase versus laser current. The maximum temperature increase caused by a 0.5 s laser pulse at various currents was measured using a thermal camera.

To characterize the noxious temperature range of *C. elegans*, we transiently raised the local temperature around a forward-moving animal by defined amounts and quantified multiple aspects of the resulting avoidance response. Heating was produced by infrared laser pulses of 0.5-s duration. Using thermal imaging, we determined that the increases in temperature above baseline (ΔT) were directly proportional to the laser power, and that the heated area encompassed the entire animal (Figure 1). We were thus able to systematically deliver thermal pulses with a ΔT between 0.4°C to 9.1°C. We recorded the behavior of each animal for 15 s in response to the thermal stimulus. From these images we measured basic features of shape such as the worm's 'skeleton', center-of-mass, head-to-tail distance, and used these measures to calculate the animal's speed and metrics of different behavioral states (see Methods).

### N2 animals exhibit dose-dependent changes in multiple aspects of the avoidance response elicited by thermal stimuli

**Figure 2:**
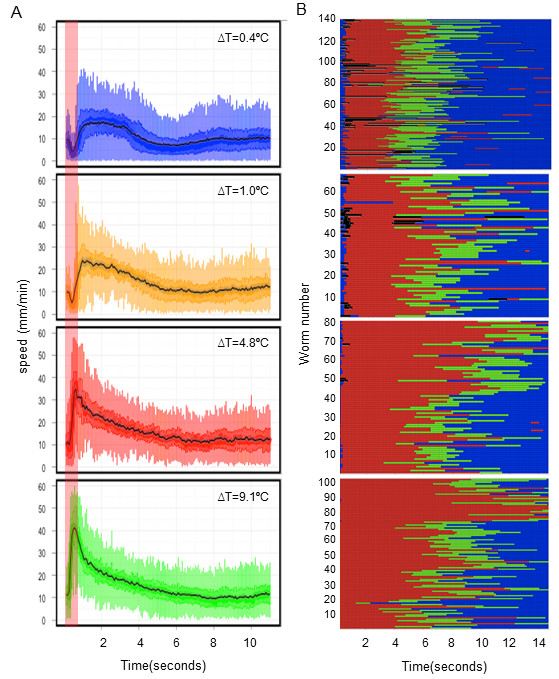
**Response of N2 animals to thermal pulses of increasing amplitude**. **(A) **Centroid speed of N2 animals plotted against time. The vertical red bar indicates duration of the pulse. The black line is the median speed profile of the animals at a given thermal pulse. The variability of speed at each time point is shown through boxplots. Temperature increases above the baseline (ΔT) as a result of thermal pulses are indicated. **(B) **Ethogram of different behavioral states of the N2 animals at indicated ΔT. The behavioral sequence of each animal over the duration of the assay (15 s) at a given ΔT is shown. Each row represents behavior of a single animal over time. Blue = forward state, red = reversal, black = pause, green = omega turns.

**Figure 3:**
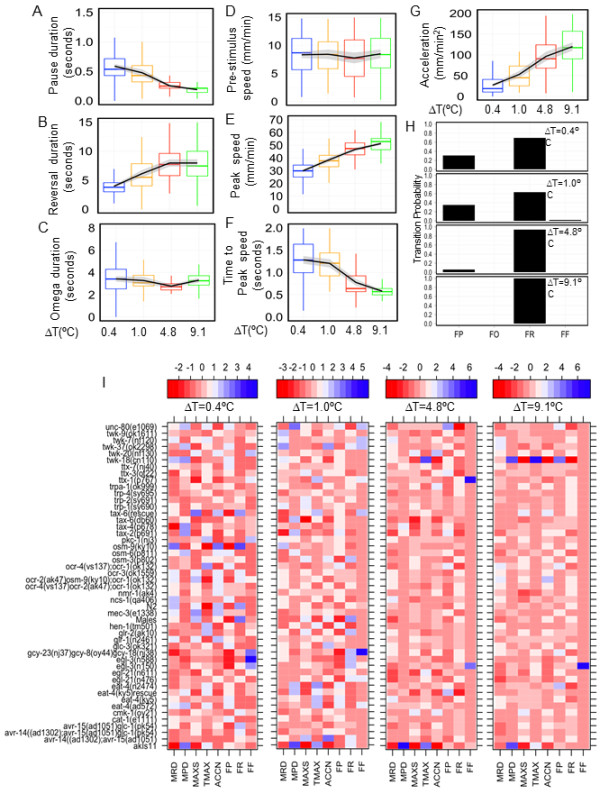
**Change in behavioral features as a function of thermal pulse of increasing amplitude**. **(A-G) **Boxplots of the corresponding behavioral feature plotted for ΔT = 0.4°C (blue), 1.0°C (orange), 4.8°C (red) and 9.1°C (green) for N2 animals. The black lines depict the trend with increasing temperature. The grey shading represents the estimated pointwise 95% confidence intervals. (A-C) Behavioral states (depicted in the ethogram of Figure 2B) plotted against ΔT. (D-G) Centroid speed related traits (depicted in Figure 2A) plotted against ΔT. **(H) **Transition probabilities between various states: FP, forward to pause; FR, forward to reversal; FO, forward to omega; FF, forward to forward. **(I) **Phenotype barcodes for alphabetically ordered 49 strains at the indicated ΔT. Each behavioral feature is Z-score normalized as described in Methods. MRD, mean reversal duration; MPD, mean pause duration; MAXS, maximum speed after stimulus; TMAX, average time to reach maximum speed; ACCN, average acceleration after stimulus.

N2 animals responded to a thermal pulse corresponding to a ΔT = 0.4°C with a stereotypical avoidance behavior that reorients the worm away from noxious stimuli. Typically, a thermal pulse applied to a forward-moving animal elicited a sequence of four behavioral states: a pause, reversal, an omega turn, and forward movement (Additional files 1 and 2, Movies S1 and S2). Increasing the amplitude of the thermal pulse did not induce any gross qualitative changes in the sequence (Figure 2). However, the duration and the speed of locomotion during specific behaviors changed proportionately as a function of the ΔT (Figure 2). From the speed (Figure 2A) and ethograms (Figure 2B), we quantified multiple behavioral parameters, including various aspects of speed changes, characteristics of different behavioral states, and the probability of switching between states at each ΔT. We found that while some features of the behavior changed with the pulse amplitude, others were stimulus independent; that is, they remained constant over an approximately 25-fold change in the amplitude of the thermal pulse (Figure 3A-H). For example, the pause duration (Figure 3A), time to respond with an increase in speed (Figure 3F) decreased as we increased the pulse amplitude. Acceleration (Figure 3G), the probability of responding with a reversal (Figure 3H) and reversal duration (Figure 3B) increased with the stimulus amplitude. However, the duration of omega turns remained constant over the entire range of stimuli (Figure 3C). We also found that the peak speed with which the worms avoided the thermal stimulus increased with the intensity of the stimulus (Figure 3E). However, the speed at the beginning of the thermal pulse stimuli did not change with an increase in amplitude of the thermal pulse (Figure 3D). We were thus able to quantify multiple aspects of the noxious, thermally induced avoidance response of *C. elegans *and identify aspects of the behavior that changed proportionately with the intensity of stimulus. We chose ΔT = 0.4°C, 1.0°C, 4.8°C and 9.1°C for further analysis, as multiple behavioral features that scaled with temperature differed significantly among these thermal pulses.

### Unique combinations of molecules define responses to different increases in temperature

**Figure 4:**
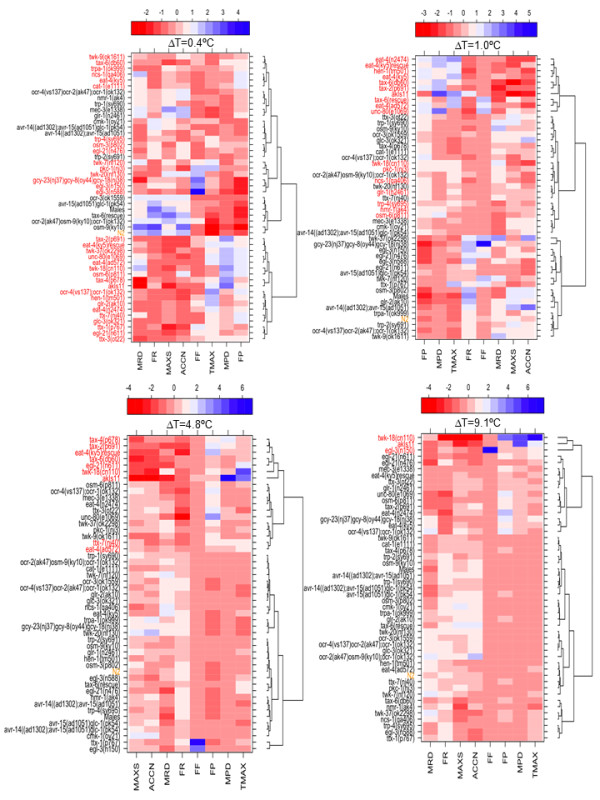
**Hierarchical clustering of the phenotype profiles of 49 strains for 8 aspects of escape behavior**. Dendrograms resulting from hierarchical clustering of the data in Figure 3I at the indicated ΔT are plotted. In the heatmaps, the strains are reordered by the dendrograms. The strains labeled with red font have a probability ≤ 0.1 to be in the same cluster as N2 (depicted with orange color) as determined by 10,000 bootstraps of their behavioral profiles at each ΔT as described in the Methods section. The behavioral features are as follows: MRD, mean reversal duration; MPD, mean pause duration; MAXS, average maximum speed after laser pulse; TMAX, mean time to reach MAXS; ACCN, mean acceleration; FP, forward to pause transition probability; FR, forward to reversal transition probability; FF, forward to forward transition probability.

The quantitative differences in the avoidance behavior that we observed with increasing intensity of the thermal stimuli can be explained by either distinct molecular mechanisms operating at different temperature ranges or a single mechanism that scales with the amplitude of the thermal pulse. If a similar set of genetically distinct strains shows defects across a broad range of ΔT, this would suggest a common molecular mechanism. On the other hand, if distinct sets of genetically different strains show defects at different temperature ranges, then this would suggest multiple molecular mechanisms. To distinguish between these possibilities, we recorded escape responses of 47 mutants in the N2 genetic background and extracted 8 behavioral features that changed with increasing intensity of the stimulus for each strain (Figure 3I). We normalized each feature, scored in different units, to a common scale. Thus at each thermal pulse, the responses of the strains were summarized as a 'phenotype barcode' of 8 features, generating a 49 strain × 8 feature matrix at each ΔT (Figure 3I). Hierarchical clustering of this strain x feature matrix identified strains that belonged to clusters different from N2 at each ΔT (Figure 4). To identify strains that are robustly different from N2, we bootstrapped the phenotype barcode data and identified strains different from N2 as ones that remained outside the N2 cluster in at least 90% of the bootstrap clusters (Figure 4, see also Methods).

**Figure 5:**
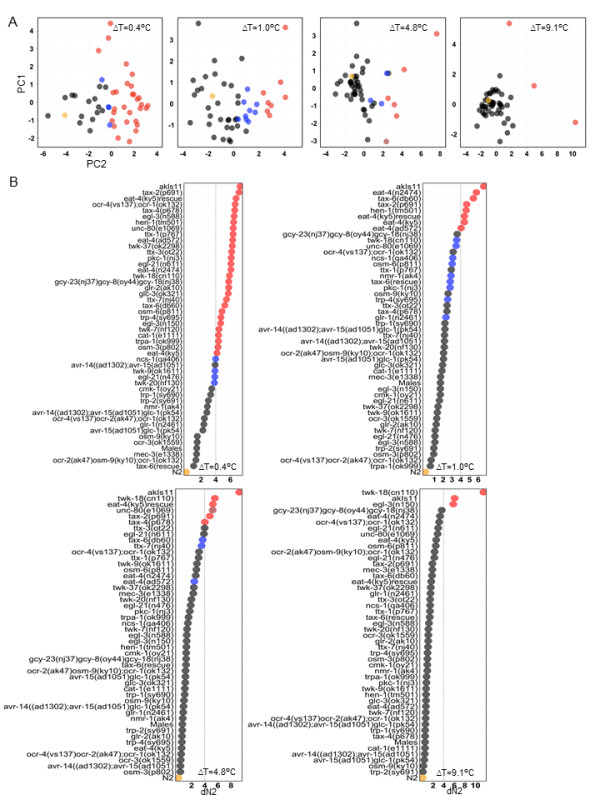
**Principal component analysis (PCA) of the 49 strains by 8 feature matrices to identify strains behaving differently from N2**. **(A) **Positions of 49 mutants in the 6-dimensional feature (principal component) space between mutants projected onto 2 dimensions by classical multidimensional scaling for the indicated ΔT. N2 is shown in orange. In red are strains that are 4 distance units away from N2 and are predicted to be different from N2 by bootstrapping. In blue are strains predicted to be different from N2 by bootstrapping and are less than 4 units away from N2 in the two-dimensional space. In black are strains that are not predicted to be different from N2 by bootstrapping and are less than 4 units away from N2 in the two-dimensional space. **(B) **Position of different strains from N2 ordered by increasing distance from N2 (dN2) calculated from the graphs in (A). The colors are as described in (A). Vertical dashed line represents 4 distance units away from N2.

**Figure 6:**
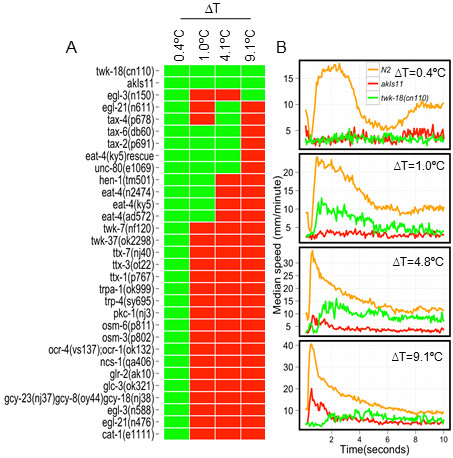
**Strains behaving differently from N2 at different ΔT**. **(A) **Heatmap of the strains predicted to be different from N2 escape response at different ΔT. Green: significantly different from N2; red: not significantly different from N2. **(B) **The changes in median speed over time of two strains, *akIs11 *and *twk-18(cn110) *predicted be different from N2 at all ΔT are shown.

As an independent means of identifying mutants that behave differently from N2 in response to a thermal pulse, we performed dimensionality reduction of the avoidance response barcodes. Principal component analysis (PCA) of 8 phenotype features produced 6 principal components that together explained approximately 95% of the variance in the avoidance responses of the 49 strains at each ΔT (Additional file 3, Table S1). We projected the strains in the six dimensional feature space onto two dimensions for each ΔT (Figure 5A, see also Methods). At all ΔT, we identified the strains that were greater than a fixed Euclidean distance away from N2 in this two-dimensional space as behaving differently from N2 (Figure 5B, see also Methods). All but one of the strains deemed different from N2 at each ΔT by this method were also predicted by bootstrapping (Figure 5B). The only exception was strain *unc-80 *at ΔT = 4.8°C, which was predicted to be different from N2 only in the PCA feature space but not by the bootstrap criteria (Figure 5A, B). Finally, we used non-parametric analysis of variance (ANOVA) on the list of the mutants predicted by either of the above criteria (PCA and hierarchical clustering) to identify a final set of strains different from N2 (see Methods). A total of 31 out of the 48 strains we tested were significantly different at either 1 (ΔT = 0.4°C) or more ΔT (Figure 6A). At each ΔT, unique sets of strains were found to be different from N2, suggesting that distinct molecular mechanisms underlie responses to noxious stimuli for temperature ranges examined.

Two strains were significantly different from N2 at all ΔT (Figure 6A; Kruskal-Wallis test, Dunn's multiple comparison *P *< 0.001), consistent with the general nature of defects in their neuromuscular system. These strains, *akIs11 *[Pnmr-1::ICE] [12] and *twk-18(cn110) *[13], are known to be defective in locomotory command interneuron and muscle function, respectively. *akIs11 *is a transgenic strain expressing human caspase (ICE) driven by the promoter of *nmr-1*, which results in the death of all the command interneurons known to be involved in mediating reversals, as well as three additional pairs of neurons. These animals are unable to execute proper reversals, and were defective at all ΔT (Figure 6B). However, these animals frequently displayed an escape response that consisted solely of omega turns without any reversals (Additional file 4, Movie S3). *twk-18(cn110) *is a temperature-sensitive gain of function mutation in a two-pore potassium channel expressed primarily in the body wall muscles [13]. This allele induces constitutive membrane hyperpolarization [13], thereby reducing excitability of the muscle at room temperature [14]. Consistent with this, animals harboring *twk-18(cn110) *were unable to elicit a wild-type escape response at any ΔT (Figure 6B).

### Thermotaxis and responses to noxious thermal pulses are genetically separable

Thermotaxis in *C. elegans *is well studied at the behavioral, neuronal and molecular level [5]. To determine whether the molecular mechanisms underlying thermotaxis are also employed during the thermal avoidance behavior, we exposed 14 strains defective in thermotaxis behavior (bold, Additional file 5, Table S2) to thermal pulses of different amplitudes. Seven of these strains (bold and underlined, Additional file 5, Table S2) were significantly impaired in responding to a ΔT = 0.4°C but exhibited normal responses to thermal pulses of larger amplitudes, suggesting that thermotaxis signals in response to small ΔT are measured through a separate pathway from thermal noxious signals in response to larger ΔT.

**Figure 7:**
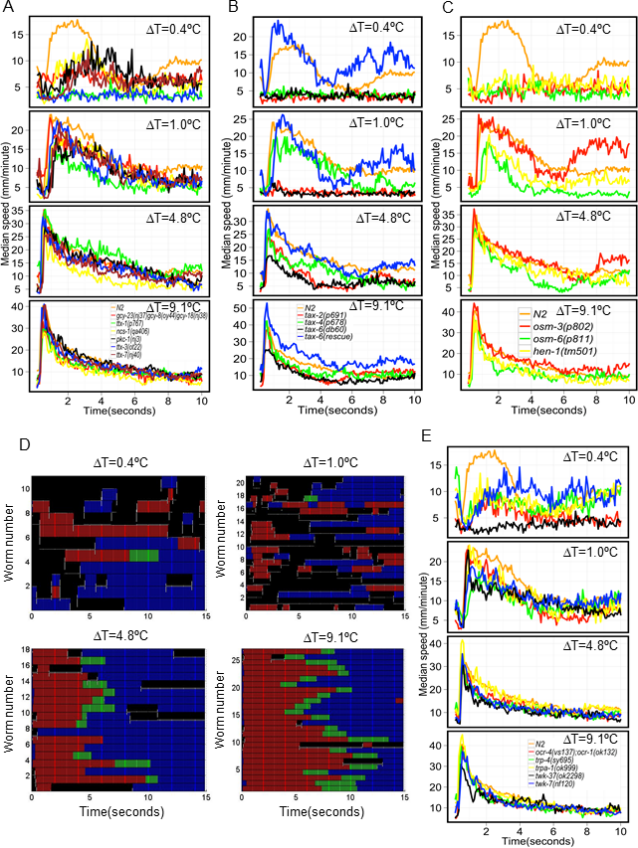
**Strains responding differently from N2 at ΔT = 0.4°C**. Changes in median speed are plotted against time. **(A-C) **Median speed profiles for strains identified to be different from N2 in Figure 6A at indicated ΔT. **(D) **Ethograms of *tax-6(db60) *at the indicated ΔT. Each row represents behavior of a single animal over time. Color scheme is same as Figure 2B. **(E) **Median speed profiles of strains harboring mutations in transient receptor potential (TRP) and two-pore K+ (TWK) channels at the indicated ΔT.

Loss of function of the homeodomain proteins, *ttx-1 *[15] and *ttx-3 *[16], required for proper development of the thermosensory neuron AFD and the interneuron AIY, respectively, results in constitutive cryophilic behavior. These mutants were defective in responses to ΔT = 0.4°C but displayed N2-like escape responses to thermal pulses of larger amplitudes (Figure 7A). We also examined the molecules in the cyclic GMP (cGMP) dependent signal transduction pathway, mutations in which results in animals with abnormal temperature preference. Loss of function mutations in the genes encoding cGMP gated channel subunits, *tax-2 *[17] and *tax-4 *[18], as well as in three genes encoding guanylate cyclases, *gcy-18*, *gcy-8 *and *gcy-23 *[19], exhibited impaired avoidance responses to ΔT = 0.4°C (Figure 7A, B). Additionally, strains harboring mutations in *tax-2 *were significantly different in their escape responses to ΔT = 1°C and 4.8°C but exhibited normal responses at ΔT = 9.1°C (Figure 7B; Kruskal-Wallis test, Dunn's multiple comparison *P *< 0.01). Animals harboring mutations in three guanylate cyclases exhibited a normal avoidance response at larger ΔT (Figure 7A), suggesting that different mechanisms may be involved in mediating responses to noxious temperature of higher magnitudes.

The homologs of calcineurin (TAX-6) [20] and protein kinase C (PKC-1) [21] are thought to negatively regulate thermosensory function of AFD, and loss of function of these genes results in constitutive thermophilic behavior. Loss of function mutants in genes encoding these molecules were also defective at ΔT = 0.4°C (Figure 7A, B). Our analysis indicated that *tax-6(lf) *mutant animals were also significantly different from N2 at ΔT = 1°C and 4.8°C but not 9.1°C (Figure 7B, D; Kruskal-Wallis test, Dunn's multiple comparison *P *< 0.01). *tax-6(lf) *mutants had multiple defects (mean reversal duration, acceleration) in their escape behavior at the lower ΔTs (Figure 7D). All defects of *tax-6 *mutant animals in thermal avoidance behavior were rescued by expressing the normal version of the gene in sensory neurons and interneurons (Figure 7B).

Loss of function of the genes *ncs-1*, encoding a neuronal calcium sensor protein [22], *cmk-1*, encoding a Ca^+2^/calmodulin-dependent protein kinase I [23] and *ttx-7*, encoding a inositol monophosphatase, required for correct localization of synapses of the interneuron RIA [24], result in abnormal thermotaxis. All three strains exhibited wild-type avoidance response at higher ΔT (Figures 6A and 7A). However, while *ncs-1 *and *ttx-7 *mutants were defective at ΔT = 0.4°C (Figure 7A), *cmk-1 *mutants behaved normally at that thermal pulse intensity.

Mutants of genes required for proper sensory cilia development, namely *osm-3 *and *osm-6 *[25], as well as of a gene encoding a secreted protein required for integration of sensory information, *hen-1 *[26], were shown to exhibit normal thermotaxis behavior. Animals harboring mutations in these genes were significantly impaired in their avoidance response to a thermal pulse of ΔT = 0.4°C (Figure 7C; Kruskal-Wallis test, Dunn's multiple comparison *P *< 0.01). Moreover, whereas the cilia defective mutants *osm-3(p802) *and *osm-6(p811) *were significantly impaired only at ΔT = 0.4°C, *hen-1(tm501) *animals were also unable to elicit a wild-type escape response at ΔT = 1.0°C (Figure 7C; Kruskal-Wallis test, Dunn's multiple comparison *P *< 0.01). These animals behaved like wild-type at ΔT = 4.8°C and 9.1°C. We have identified molecules (*osm-3, osm-6 and hen-1*) that are involved in responses to noxious thermal stimuli but are not required for thermotaxis. We also identified strains that are not defective in avoidance of thermal pulses but exhibit abnormal thermotaxis behavior (for example, *cmk-1*). Thus, thermotaxis and the escape response to a thermal pulse of ΔT = 0.4°C are genetically separable.

### TRP and two-pore K+ (TWK) channels are required for mediating normal avoidance responses to ΔT = 0.4°C

Although the major thermosensors of invertebrates are ion channels of the TRP family [2], such channels have a relatively mild role in mediating responses to thermal stimuli in *C. elegans*. For example, mutations in the TRPV (ion channels known to be activated by heat in mammals) homolog of *C. elegans*, *osm-9 *[27], have been shown to exhibit mild defects in the thermal avoidance response [7,8]. However, the TRPA ion channel homolog of *C. elegans *has been shown to be required for acute cold sensation [28]. To test the function of TRP channels in sensing different intensities of noxious stimuli, we examined the avoidance response of nine strains with mutations in different TRP channels elicited by thermal pulses with ΔT of 0.4°C, 1.0°C, 4.8°C and 9.1°C. Three of these mutants, *trpa-1 *(TRPA), *ocr-1;ocr-4 *(TRPV) and *trp-4 *(TRPN) [29], were defective in avoidance responses to ΔT = 0.4°C, but their responses did not differ significantly from N2 at the higher ΔT (Figure 7E; Kruskal-Wallis test, Dunn's multiple comparison, *P *< 0.01). However, we did not detect any major defect in the behavior of animals harboring mutations in the gene encoding TRPV channel subunit homolog OSM-9, which is required for sensation of noxious thermal stimuli (approximately 43°C) in mammals.

TWK channels have been implicated in defining temperature thresholds and ranges of activation of thermosensory neurons in mammals [30]. In *C. elegans*, there are approximately 40 genes encoding TWK channels [31]. We examined the effect of loss-of-function mutations in four TWK channels on the thermally induced avoidance responses. Animals harboring loss of function mutations in either *twk-7 *or *twk-37 *were impaired in their avoidance response at ΔT = 0.4°C but not at higher ΔT (Figure 7E).

### Glutamatergic neurotransmission is essential for responses to ΔT = 0.4°C and 1.0°C

**Figure 8:**
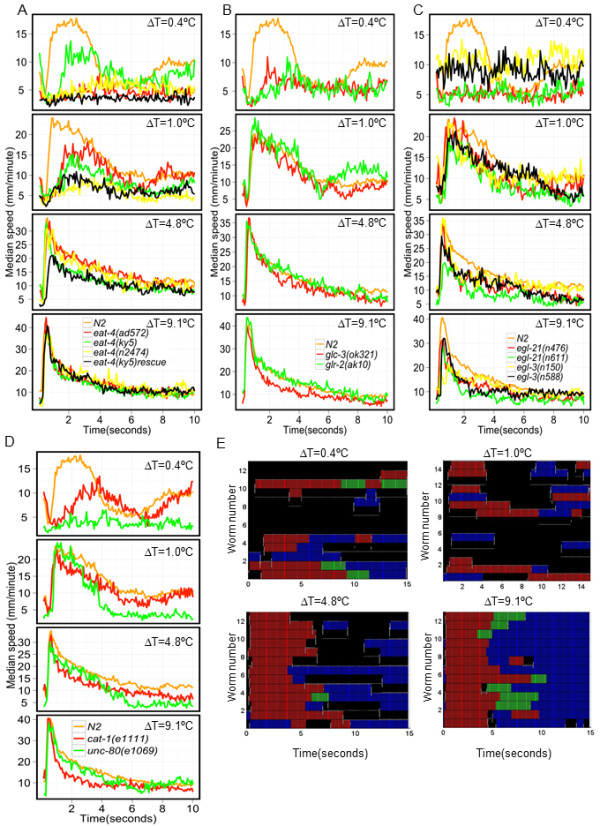
**Role of neurotransmitter signaling in sensation of noxious thermal stimuli**. Changes in median speed are plotted against time for **(A) **different alleles of gene encoding glutamate transporter (*eat-4) *and transgenic glutamate transporter mutant (*eat-4(ky5) *expressing EAT-4 under the *odr-3 *promoter (*eat-4(ky5) *rescue). **(B) **Animals harboring mutations in glutamate gated channels, *glr-2 *and *glc-3*. **(C) **Strains defective in neuropeptide processing pathway. Speed profiles of animals harboring mutations in *egl-3 *and *egl-21 *are plotted at the indicated ΔT. **(D) **Animals harboring loss-of-function mutations in *cat-1 *and *unc-80 *genes. **(E) **Ethograms of *unc-80(e1069) *at the indicated ΔT. Each row represents behavior of a single animal over time. Color scheme is same as Figure 2B.

Glutamatergic neurotransmission has been reported to be essential for normal thermotaxis [32], as well as for avoidance responses induced by noxious thermal stimuli [7]. Thus it was likely that glutamate would play a role in the thermal avoidance response in our thermal pulse assays. To test this hypothesis, we examined three loss-of-function alleles of the gene encoding the vesicular glutamate transporter (*eat-4*) that concentrates glutamate onto synaptic vesicles. All three alleles were defective in eliciting a wild-type response to a thermal pulse corresponding to ΔT = 0.4°C and 1°C, but were not significantly different from N2 at larger ΔT (Figure 8A). Transgenic expression of functional EAT-4, driven by the *odr-3 *promoter, failed to rescue the defects of *eat-4(ky5) *at ΔT = 0.4°C and 1.0°C and induced a significantly impaired escape response at ΔT = 4.8°C (Figure 8A; Kruskal-Wallis test, Dunn's multiple comparison *P *< 0.01). *odr-3 *drives expression in a subset of neurons (AWA, AWB, AWC and ASH) in which EAT-4 is normally expressed. Our results suggest that additional neurons beyond the ones defined by the expression driven by *odr-3 *promoter are required to elicit a proper escape response at ΔT = 0.4°C and 1.0°C. The abnormal phenotype at ΔT = 4.8°C and N2-like avoidance response at ΔT = 9.1°C of this strain suggest involvement of distinct sets of neurons contributing to avoidance responses at these thermal pulse amplitudes.

We also analyzed the thermally induced escape responses of animals harboring mutations in the genes encoding the 2-amino-3-(3-hydroxy-5-methyl-isoxazol-4-yl)propanoic acid (AMPA) and *N*-methyl-D-aspartate (NMDA) classes of glutamate receptors, as well as glutamate-gated chloride channels. We detected significantly impaired escape responses induced by ΔT = 0.4°C in strains harboring mutations in the glutamate-receptor subunit *glr-2 *and the glutamate-gated chloride channel *glc-3 *(Kruskal-Wallis test followed by Dunn's multiple comparison). Escape responses of these strains to thermal pulses resulting in larger ΔT were indistinguishable from N2 (Figure 8B). However, we observed that *eat-4 *mutants were defective in their avoidance response at ΔT = 1.0°C (Figure 8A) suggesting that different combinations of glutamate-gated channels were responsible for mediating effects of glutamatergic neurotransmission at ΔT = 1.0°C. Additionally, defects in catecholaminergic neurotransmission due to a mutation in the vesicular monoamine transporter, *cat-1*, resulted in abnormal avoidance responses only at ΔT = 0.4°C (Figure 8D). Thus response to a thermal stimulus of ΔT = 0.4°C requires participation of both glutamatergic and catecholaminergic neurotransmission.

### Neuropeptides define escape responses to a subset of thermal pulse stimuli

Neuropeptide signaling was shown to be required for avoidance of noxious thermal stimuli [7,8]. We examined thermally induced avoidance responses of mutants harboring loss of function of genes encoding proprotein convertase (*egl-3) *and carboxypeptidase (*egl-21*) [33] that are required for efficient neuropeptide processing. We found that animals carrying loss-of-function alleles of these genes were defective in responding to a ΔT = 0.4°C. Interestingly, all neuropeptide-processing-impaired strains were able to elicit an avoidance response similar to N2 at ΔT = 1.0°C. However, animals harboring loss-of-function mutations in *egl-21 *and *egl-3 *were defective at ΔT = 4.8°C and ΔT = 9.1°C, respectively. This difference probably reflects the penetrance of the different alleles of *egl-21 *and *egl-3 *with respect to the thermal avoidance behavior (Figure 8C). We conclude that whereas glutamate and neuropeptide signaling is required for responses to ΔT = 0.4°C, neuropeptides but not glutamate are dispensable for responses at ΔT = 1.0°C. For higher ΔT, we found neuropeptide but not glutamate involvement in mediating different aspects of the thermal avoidance behavior. Taken together, these results suggest that distinct combinations of glutamatergic, catecholaminergic and peptidergic signaling shape avoidance responses at different intensities of noxious thermal stimuli. However, the lack of consistent responses for multiple alleles of the same gene precludes us from a definitive conclusion about the involvement of neuropeptides at higher ΔT. Nevertheless, taken together, these data suggest that distinct combinations of neurotransmitter systems operate at ΔT = 0.4°C, 1.0°C, 4.8°C and 9.1°C to give rise to a qualitatively similar stereotypical behavioral response.

## Discussion

Most studies on noxious temperature sensation of *C. elegans *have been restricted to responses resulting from a rise in temperature of approximately 10°C to 15°C above the baseline [7,8,10]. However, between thermotaxis, where animals sense an approximately 0.05°C rise in temperature above the baseline, and noxious thermal stimuli, there is an approximately 200-fold change in temperature. In this study, we explored how an animal responds to changes in the noxious quality of thermal stimuli. We considered the noxious quality of the thermal stimuli to be proportional to the amplitude of the thermal pulses, and observed quantitative changes in the avoidance response with increasing amplitude of the stimulus. Quantitative changes in the avoidance response could be due to either a single molecular mechanism proportionately changing with intensity of the stimulus, or multiple separate molecular mechanisms operating in distinct temperature ranges, which converge onto a qualitatively similar avoidance response. Through use of multiple mutants, we provide evidence of a molecular partitioning of thermosensation in the temperature range of *C. elegans *that elicits an escape behavior. This property is similar to temperature sensation in mammals, where sensations of distinct temperature ranges are mediated by different sets of molecules. However, compared to mammals, discrimination of noxious temperature in *C. elegans *appears to be over a much narrower range.

Within our candidate list of strains, we observed that avoidance responses at lower ΔT involved a larger number of molecules. With increasing amplitude of the thermal pulse, fewer molecules were essential for generating a normal avoidance response. It is possible that multiple redundant pathways engage at larger ΔT to generate the thermally induced avoidance response. Consistent with the possibility of involvement of multiple redundant pathways in sensing noxious thermal stimuli, Glauser *et al*. [8] reported parallel involvement of a neuropeptide and a TRP channel. Additionally, Liu *et al*. [10] suggested that multiple neurons (AFD and FLP in the head) and molecules (cyclic nucleotide-gated (CNG) and TRP channels) act in parallel to induce avoidance to noxious thermal stimuli. However, some noxious-avoidance-defective mutants reported by Liu *et al*. and Glauser *et al*., for example, the TRPV and cyclic-nucleotide gated channel mutants, behaved normally in our assay for the largest stimulus ΔT equal to approximately 9.1°C. The assay of Liu *et al*. involves ΔT equal to approximately 16°C with an absolute peak temperature of 38°C, whereas our assay corresponds to ΔT equal to approximately 9.1°C with an absolute peak temperature of 33°C. Also we heated the entire worm body as opposed to just the head or tail region of the worm, and we used a much steeper ramp rate. It is possible that there exists an additional TRPV and CNG channel-dependent temperature threshold beyond the ones we tested. Further studies will be required to test this hypothesis, but it is clear that in the noxious temperature range of *C. elegans*, there exist multiple distinct molecularly determined temperature ranges that are quantitative determinants of the avoidance response. Although we used well characterized mutants in our study, the lack of transgenic rescue experiments, except for *tax-6*, prevents us from distinguishing whether the mutations in the genes themselves or additional mutations in the mutant background are shaping the avoidance response. This is perhaps less of a drawback for those cases where multiple alleles gave rise to a similar phenotype. This limitation, however, does not change our conclusion of distinct molecular combinations acting in different temperature regimes as we have compared the same set of strains in response to the same thermal stimuli.

Responses to noxious temperature have previously been studied by scoring only one aspect of the avoidance behavior, for example, the fraction of animals responding by reversal. However, as we have shown, multiple aspects of the escape response change quantitatively with increasing intensity of the noxious stimuli. Focusing on one aspect of the response could result in some mutants being misclassified as not significantly different from N2. For example, a similar fraction of *eat-4(ky5) *and N2 animals responded to ΔT = 0.4°C by a reversal, however the time it took to initiate reversals was significantly longer for *eat-4(ky5) *animals. The multidimensional measure of worm behavior allowed us to better characterize the effect of genetic manipulations on the escape response.

Our analyses also provided new insights into the effect of molecules on the motor output of the escape behavior. For example, *akIs11 *animals frequently displayed an escape response that consisted solely of omega turns without any reversals for all ΔT (Additional file 4, Movie S3), suggesting that execution of reversals and omega turns is encoded by distinct neurons, in agreement with earlier studies [34]. In fact, even though *akIs11 *did not perform a reversal, the duration of time before the observed omega turn increased with the ΔT, just as the reversal time increases with the ΔT for N2 worms, suggesting that parallel pathways process the thermal stimulus information that generates the duration of reversals or the timing of omega turns. Additionally, animals harboring mutations in *unc-80 *(required for expression and localization of nematode calcium channel (NCA) type calcium channels) [35] were defective at all but the highest ΔT. These mutants display a 'fainter' phenotype in which reversals are followed by an abrupt stop rather than by an omega turn, suggesting a block in switching from the reversal to omega turns. At lower ΔT, the majority of animals moved backwards and paused instead of making an omega turn (Figure 8D, E). This defect was largely suppressed when animals harboring mutations in *unc-80 *were exposed to a thermal pulse of ΔT = 9.1°C (Figure 8E). This result suggests that the block from reversals to the subsequent motor pattern is bypassed by a thermal pulse amplitude somewhere between ΔT = 4.8°C and ΔT = 9.1°C.

Analysis of the effect of genetic manipulations at a genome-wide scale, for example, through systematic RNAi, should provide a comprehensive understanding of the neuronal and molecular basis of a simple sensorimotor transformation such as the one introduced here. Similar studies incorporating multiple aspects of behavior have been used to classify different mutants or to screen the effects of drugs [36-39]. However, to our knowledge, the use of high-content data to understand the neurogenetic basis of a sensorimotor transformation is novel. High-content phenotyping and the behavioral barcoding approach, as exemplified by our study, will improve our understanding of how sequences of behavior are integrated at the neuronal and molecular level.

## Conclusions

We have developed a novel laser-heating assay to comprehensively study the behavioral response of individual *C. elegans *to precise amounts of thermal stimulus. The multidimensional behavioral profile of worms was quantified using computer-based imaging and data analysis. A candidate library of 47 strains was studied to determine which sets of molecules are involved in thermal sensation at different stimulus intensities. The resulting data revealed that a number of genes involved in thermotaxis are not necessary for thermal nociception, and that different sets of molecules are involved in the thermal response to different thermal stimulus intensities.

## Methods

### Strains

All strains used in this study were maintained under standard nematode culture conditions [40]. Unless mentioned otherwise all animals assayed were of the hermaphrodite sex. The strains and the number of worms analyzed in the calculation of various behavioral metrics are depicted in Additional file 5, Table S2. The data and the images generated for analyses of the avoidance behavior for the mutant strains are available from the authors upon request.

### Behavioral assays

Unless otherwise mentioned mid to late L4 stage worms were picked on 6 cm standard nematode growth media (NGM) plates with agar approximately 24 h before the assay. Assay plate preparation: 10 cm plates containing 10 ml of agar medium (17 g of agar (Difco, Detroit, MI, USA), 2.7 g of Bactopeptone (Difco), 0.55 g of Tris base (Sigma, St. Louis, MO, USA), 500 μl of 1(M) Tris HCl (Sigma), 2.0 g of NaCl (Fisher Scientific, Pittsburgh, PA, USA), and 1 ml of ethanol containing 5 mg/ml cholesterol (Sigma), per l H_2_O). Plates were stored at 4°C and used within 2 weeks. A total of 100 μl of *Escherichia coli *(op50) approximately 0.6 to 0.8 OD was spread evenly on the agar plates at least 16 h prior to the assay. On the day of the assay single worms were transferred to the agar plates seeded with bacteria and kept at 20°C for at least 10 minutes. The worms were then subjected to thermal stimulus as described below.

### Thermal stimulus assay

Worms were imaged using a Leica MZ16APO stereomicroscope and a Basler firewire CMOS camera (A602fm; Basler, Ahrensburg, Germany). A collimated beam with a 1/e diameter of 1.50 mm from a 1,440 nm diode laser (FOL1404QQM; Fitel, Peachtree City, GA, USA) was positioned to heat the area covering the worm. The diode laser was driven with a commercial power supply and controller (LDC 210B and TED 200C; Thorlabs, Newton, NJ, USA). A custom program written in LabVIEW (National Instruments, Austin, TX, USA) was used to control the firing, power, and duration of the infrared (IR) laser, while simultaneously recording images of the crawling worm for 15 s at 14 or 28 Hz. Images were processed offline using custom programs written in LabVIEW and MATLAB (Mathworks, Natick, MA, USA). A thermal camera (ICI 7320, Infrared Camera Inc., Beaumont, TX, USA) was used to measure the temperature of the agar when heated by the IR laser. We were able to generate thermal pulses with a temperature change ranging from ΔT = 0.4°C to 9.1°C with ramp rates ranging from 0.8°C/s to 18°C/s respectively. These ΔT were measured from the baseline temperature (or room temperature), which was typically approximately 20°C.

### Behavioral quantification

The behavior of the worms upon response to a thermal stimulus was quantified in multiple ways. Speed profile of each worm over time was generated by a custom written program in MATLAB and LabVIEW that monitored the position of the Center of Mass (COM) of the worm image. The behavioral states of the worm were identified using programs to automatically and semiautomatically identify stereotyped worm behaviors from sequence of captured images. All analysis described below was performed separately on the 14 fps and 28 fps data. Once processing was complete, the 14 fps data was upsampled, converted from frames to time (in seconds) and combined with the 28 fps data to produce the final results.

### Preprocessing

The images were run though a skeletonization algorithm written in MATLAB to find the endpoints of the worm in each frame. The curvature of the perimeter was used to identify the head and tail, since the maximum curvature is found at the tail. The endpoints and centroid were tracked frame by frame for each dataset, and the automatic detection of events in each frame is outlined below.

It is assumed that the worm is going FORWARD at the moment the thermal stimulus is applied. All frames at the beginning of the behavioral quantification are marked as FORWARD, unless they meet the criteria of another behavioral marker, namely an OMEGA TURN, a REVERSAL, or a PAUSE.

### Criteria for an OMEGA TURN

(a) When the head to tail distance is less than half of the maximum head to tail distance, the frame is tentatively flagged as the start of an omega turn. (b) If the frame flagged in (a) also corresponds to a COM speed of < 1 pixel/frame, this frame is flagged as the start of an omega turn. (c) The end of the omega turn is flagged when the head to tail distance is greater than half of the maximum head to tail distance. (d) All frames between the start and end frames are flagged as OMEGA TURN.

### Criteria for a REVERSAL: first case

The dataset contains an omega turn. In this case, (a) for frame number 1 to the start of the omega turn, the centroid trajectory is sampled at every 2 frames. If the turning angle is greater than 120 degrees, the frame is flagged as the start of a reversal. (b) The end of the reversal is the start of the omega turn.

### Criteria for a REVERSAL: second case

The dataset does not contain an automatically detected omega turn. In this case, (a) for all frames in the data set, the centroid trajectory is sampled at every 2 frames. If the turning angle is greater than 120 degrees, the frame is flagged as the start of a reversal. (b) The end of the reversal is flagged as when there is another turning event greater than 120 degrees in some frame after the frame flagged in (a). All frames between these two frames are marked as the worm is in a reversal. The reversal detection then repeats to see if there are any other reversals after the first detected reversal.

### Criteria for a PAUSE

If the COM speed is less than 7 μm per frame, the head to tail distance is concurrently at least three-quarters of the maximum, and these conditions are both met for three consecutive frames, the worm is said to be in a pause state until the COM speed becomes greater than 7 μm per frame.

The aforementioned thresholds for the behavioral flags were determined using N2 data. For strains with severe locomotory defects, thresholds were adjusted on a case-by-case basis. The user verified the automatic flagging of these behavioral states and changed any erroneous/missed flags by eye.

### Statistical analyses

Statistical analyses were performed in the R programming language [41] using the following add-on libraries: ClassDiscovery [42], Lattice [43], RColorBrewer [44], and ggplot2 [45].

### Generation of behavioral barcodes

We generated behavioral profiles of 48 strains in response to thermal pulses corresponding to ΔT = 0.4°C, 1.0°C, 4.8°C and 9.1°C. Initially we characterized behavioral responses of the reference strain N2 at these stimuli. There were five features that consistently changed as a function of increasing amplitude of the thermal pulse and thus we used these for further analysis. We quantified four additional behavioral responses of a forward moving worm when presented with a thermal pulse by calculating the transition probabilities FF, FR, FO, and FP (that is, forward to forward, reversal, omega turn, and pause respectively). FF captures worms not responding to the thermal stimulus, that is, continuing their forward motion. Any three of these features would distinguish worms not reacting to the thermal stimuli from ones that do react. We extracted 8 features (Figure 3I) from escape responses of the 47 additional strains. Thus at each ΔT we generated a matrix of 48 strains by 8 features. As these features, *f*, were measured in different units we normalized them across all strains to a Z-score, Z = [*f *- mean (*f*)]/SD(*f*), where SD is the standard deviation of a given feature. We generated four such matrices shown in Figure 3I, where each column corresponds to a feature with mean = 0 and SD = 1.

### Hierarchical clustering to identify strains behaving differently from N2

To identify strains that behaved differently from the reference strain N2 at each ΔT, we used agglomerative hierarchical clustering. We used the Euclidean distance to define the proximity of strains to one another, and used Ward's method to define the distance between two clusters, which determines the cluster structure by minimizing the sum of squares when two clusters are joined. Finally we presented the results of the clustering as a dendrogram as shown in Figure 4. The add-on package Lattice in the R statistical language platform was used to generate the dendrograms. Hierarchical clustering procedures such as the ones we have used will always generate clusters from any given dataset. To determine the robustness of the clusters generated by the method above, we used a bootstrapping method [46] where we repeatedly resampled the 48 strains with replacement and obtained the number of times each pair of samples ended up in the same branch of the dendrogram. We performed 10,000 bootstrap clustering generating 4 clusters for each round of bootstrapping. Next we determined the fraction of time each strain belonged to the same cluster as N2. Finally, we assigned strains that remained outside the N2 cluster in 90% of the 10,000 bootstrap clusters as different from N2 in their behavioral barcode. These operations were performed using the R add-on package ClassDiscovery. We performed the above clustering procedure separately on the four matrices shown in Figure 3I. The results of the bootstrapping are depicted in Figure 4.

### Principal components analysis to identify strains behaving differently from N2

The behavioral metrics we used in our analysis are subjectively defined and hence may be correlated with each other. To use independent uncorrelated behavioral metrics in identifying strains different from N2 we used PCA to reduce the dimensionality of the behavioral features. We found that at each ΔT, ≥ 95% of the variance in behavioral metrics could be explained by the first six principal components (Additional file 3, Table S1). We effectively transformed our raw behavioral metrics into a set of six principal components that defined the behavior of the strains in response to the thermal pulses. Next we projected the proximity relationships among 48 strains in this 6-dimensional feature space onto 2 dimensions by using classical multidimensional scaling (MDS). MDS is a procedure for mapping between high dimensional and low dimensional space and helps visualization of the relationships in a lower dimensional space. Essentially it finds a configuration of data points in a low dimensional space (two dimensions in our case) that best captures the proximity of points in a higher dimensional space (six dimensional in our case). After generating a scatterplot by MDS (Figure 5A) we calculated the distance of each of the 48 strains from the reference strain N2. This also allowed us to compare the results with that of hierarchical clustering (red and blue dots in Figure 5A). To select a criteria for strains different than N2, a distance d_0 was chosen that separated the three strains shown to be different than N2 at ΔT = 9.1°C using hierarchical clustering and ANOVA. Then we assigned any strain that was ≥ d_0 away from N2 as behaving differently from N2 at all ΔT. The distances that we measured and plotted in Figure 5B were thus a quantification of the visualizations in Figure 5A.

### ANOVA

The above two methods of predicting strains that behave differently from N2 at a given ΔT uses the mean of behavioral features and hence does not take into account the underlying distribution of the data. To verify the strains predicted to be different from N2 at each ΔT by the above two methods, we performed Kruskal-Wallis test followed by Dunn's multiple comparison test [47] for each of the behavioral metrics. We considered two strains to be significantly different at a confidence level of *P *≤ 0.01 for at least one of the behavioral metrics we analyzed.

The speed profiles we present in the Figures 6, 7 &8 are a global summary of the parameters we used for the analyses to determine the significance of difference between two strains. The significance of the differences in behavioral metrics (quantified from the speed profiles) of a mutant strain from N2 was determined by Kruskal-Wallis test followed by Dunn's multiple comparison. We have referred to mutant strains as 'significantly' different from N2 in the Results section, if at least one of the metrics derived from the speed profiles differed significantly from N2 at a confidence level of *P *< 0.01.

## Competing interests

The authors declare that they have no competing interests.

## Authors' contributions

RG and WSR conceived the study and designed the experiments. RG and AM analyzed the data with suggestions by WSR and LK. RG, WSR, and LK wrote the manuscript. All authors read and approved the final manuscript.

## Supplementary Material

Additional file 1 **Movie S1**. Escape behavior of a N2 animal to ΔT = 0.4°C. Click here for file

Additional file 2 **Movie S2**. Escape behavior of a N2 animal to ΔT = 9.1°C. Click here for file

Additional file 3 **Table S1**. Proportion of the variance explained by the first six principal components. At each ΔT this amounts to ≥ 0.95. Click here for file

Additional file 4 **Movie S3**. Escape behavior of an *akIs11 *animal to ΔT = 9.1°C. Click here for file

Additional file 5 **Table S2**. List of strains for thermal stimulus assay and the number of animals used to extract the behavioral parameters. Click here for file
