# Supplementary material for: Multiparameter behavioral profiling reveals distinct thermal response regimes in Caenorhabditis elegans
Source: BMC Biol. 2012 Oct 31;10:85. doi: 10.1186/1741-7007-10-85 (PMC3520762; doi:10.1186/1741-7007-10-85)
Supplement: Additional file 3 — Table S1. Proportion of the variance explained by the first six principal components. At each ΔT this amounts to ≥ 0.95. [file 1741-7007-10-85-S3.DOCX]

Table 1: Proportion of variance explained by first six principal components

| ∆T | PC1 | PC2 | PC3 | PC4 | PC5 | PC6 |
| --- | --- | --- | --- | --- | --- | --- |
| 0.4ºC | 0.54 | 0.21 | 0.1 | 0.06 | 0.05 | 0.02 |
| 1.0ºC | 0.48 | 0.18 | 0.12 | 0.1 | 0.06 | 0.04 |
| 4.8ºC | 0.47 | 0.19 | 0.13 | 0.09 | 0.06 | 0.05 |
| 9.1ºC | 0.52 | 0.16 | 0.13 | 0.1 | 0.06 | 0.03 |
