# Supplementary material for: Multiparameter behavioral profiling reveals distinct thermal response regimes in Caenorhabditis elegans
Source: BMC Biol. 2012 Oct 31;10:85. doi: 10.1186/1741-7007-10-85 (PMC3520762; doi:10.1186/1741-7007-10-85)
Supplement: Additional file 5 — Table S2. List of strains for thermal stimulus assay and the number of animals used to extract the behavioral parameters. [file 1741-7007-10-85-S5.DOCX]

Table 2: Number of animals analyzed for the indicated strains

| Strain | ∆T =0.4ºC | ∆T =1.0ºC | ∆T =4.8ºC | ∆T =9.1ºC |
| --- | --- | --- | --- | --- |
| *akIs11* | 14 | 16 | 30 | 32 |
| *avr-14((ad1302);avr-15(ad1051)* | 15 | 16 | 12 | 19 |
| *avr-14((ad1302);avr-15(ad1051)glc-1(pk54)* | 21 | 19 | 21 | 22 |
| *avr-15(ad1051)glc-1(pk54)* | 21 | 16 | 15 | 16 |
| *cat-1(e1111)* | 22 | 21 | 20 | 20 |
| ***cmk-1(oy21)*** | 18 | 18 | 11 | 18 |
| ***eat-4(ad572)*** | 19 | 20 | 22 | 11 |
| ***eat-4(ky5)*** | 20 | 25 | 22 | 21 |
| *eat-4(ky5)rescue* | 18 | 22 | 14 | 15 |
| ***eat-4(n2474)*** | 14 | 21 | 10 | 10 |
| *egl-21(n476)* | 24 | 21 | 23 | 28 |
| *egl-21(n611)* | 15 | 15 | 15 | 16 |
| *egl-3(n150)* | 20 | 15 | 23 | 35 |
| *egl-3(n588)* | 10 | 14 | 12 | 14 |
| ***gcy-23(nj37)gcy-8(oy44)gcy-18(nj38)*** | 27 | 11 | 12 | 10 |
| ***glc-3(ok32****1)* | 16 | 16 | 14 | 14 |
| *glr-1(n2461)* | 20 | 23 | 16 | 15 |
| *glr-2(ak10)* | 12 | 11 | 14 | 14 |
| *hen-1(tm501)* | 11 | 14 | 12 | 16 |
| *mec-3(e1338)* | 12 | 15 | 14 | 13 |
| N2 | 180 | 70 | 80 | 107 |
| ***ncs-1(qa406)*** | 16 | 14 | 15 | 15 |
| *nmr-1(ak4)* | 21 | 17 | 12 | 12 |
| *ocr-2(ak47)osm-9(ky10);ocr-1(ok132)* | 16 | 15 | 15 | 11 |
| *ocr-3(ok1559)* | 19 | 22 | 26 | 20 |
| *ocr-4(vs137);ocr-1(ok132)* | 13 | 15 | 16 | 16 |
| *ocr-4(vs137)ocr-2(ak47);ocr-1(ok132)* | 31 | 22 | 20 | 20 |
| *osm-3(p802)* | 14 | 14 | 13 | 14 |
| *osm-6(p811)* | 16 | 16 | 16 | 18 |
| *osm-9(ky10)* | 37 | 15 | 21 | 45 |
| ***pkc-1(nj3)*** | 10 | 12 | 12 | 12 |
| ***tax-2(p691)*** | 10 | 12 | 16 | 20 |
| ***tax-4(p678)*** | 17 | 10 | 15 | 14 |
| ***tax-6(db60)*** | 11 | 21 | 17 | 25 |
| *tax-6(rescue)* | 14 | 15 | 16 | 16 |
| *trp-1(sy690)* | 15 | 24 | 26 | 21 |
| *trp-2(sy691)* | 21 | 21 | 20 | 20 |
| *trp-4(sy695)* | 27 | 20 | 21 | 23 |
| *trpa-1(ok999)* | 20 | 11 | 20 | 25 |
| ***ttx-1(p767)*** | 14 | 18 | 19 | 16 |
| ***ttx-3(ot22)*** | 14 | 14 | 12 | 12 |
| ***ttx-7(nj40)*** | 18 | 17 | 17 | 16 |
| *twk-18(cn110)* | 17 | 17 | 16 | 17 |
| *twk-20(nf130)* | 15 | 15 | 15 | 15 |
| *twk-37(ok2298)* | 14 | 15 | 16 | 15 |
| *twk-7(nf120)* | 17 | 16 | 14 | 14 |
| *twk-9(ok1611)* | 16 | 15 | 17 | 17 |
| *unc-80(e1069)* | 13 | 15 | 13 | 13 |
